# Pathological complete response after percutaneous isolated hepatic perfusion in hepatocellular carcinoma with portal vein tumor thrombosis: a case report

**DOI:** 10.1186/s40792-016-0178-x

**Published:** 2016-05-26

**Authors:** Keisuke Arai, Takumi Fukumoto, Motofumi Tanaka, Kaori Kuramitsu, Masahiro Kido, Hisoka Kinoshita, Taku Matsumoto, Hirochika Toyama, Sadaki Asari, Tadahiro Goto, Tetsuo Ajiki, Yonson Ku

**Affiliations:** Division of Hepato-Biliary-Pancreatic Surgery, Department of Surgery, Graduate School of Medicine,, Kobe University, 7-5-2, Kusunoki-cho, Chuo-ku, Kobe, Hyogo 650-0017 Japan

**Keywords:** Hepatocellular carcinoma, Portal vein tumor thrombosis, Percutaneous isolated hepatic perfusion, Preoperative chemotherapy

## Abstract

**Background:**

Although the effectiveness of perioperative adjuvant therapy in the treatment of hepatocellular carcinoma (HCC) has been investigated, the efficacy of preoperative therapy is unclear. Herein, we report a case of pathological complete response after percutaneous isolated hepatic perfusion (PIHP) for HCC involving portal vein tumor thrombosis (PVTT).

**Case presentation:**

A 77-year-old woman was referred to our institute with a liver mass detected on a routine health screening. Computed tomography revealed a 28 × 25 mm HCC in the left lobe of the liver and a tumor thrombus in the left and right portal branches (T4N0M0, stage IVA). The patient received a single dose of preoperative PIHP with doxorubicin plus mitomycin C, without severe toxicity. After the chemotherapy, she underwent extended left hepatic lobectomy and thrombectomy of the PVTT. No cancer cells were detected during histopathological analysis, indicating pathological complete response. She remained relapse-free 12 months after the surgery.

**Conclusions:**

We experienced a case of pathological complete response after preoperative PIHP with doxorubicin plus mitomycin C for HCC involving PVTT.

## Background

Hepatocellular carcinoma (HCC) is the sixth most common cancer and third leading cause of cancer-related deaths worldwide [1]. Surgical resection is the most promising treatment for long-term survival although only 10–37 % of the cases with HCC are eligible for surgical resection because of the extent of disease or cirrhosis-related liver dysfunction [2]. Portal vein tumor thrombosis (PVTT) is a common complication of HCC associated with a poor prognosis [3]. For the treatment of HCC involving PVTT, according to Japanese Clinical Practice Guidelines for Hepatocellular Carcinoma 2013, liver resection may be selected for patients with liver damage of grade A along with three or less HCC [4]. However, the use of curative surgical resection is controversial because it is associated with a high recurrence rate [5–9]. Therefore, effective multimodality treatment approaches should be explored to improve the overall and survival outcomes. Percutaneous isolated hepatic perfusion (PIHP) is a form of high-dose regional chemotherapy that enables the administration of cytotoxic agents at a dose of up to 10 times the maximally tolerated dose, while reducing exposure of the entire body to the major adverse effects of the therapeutic agents [10]. We showed in our previous studies that PIHP is safe and effective in the treatment of local tumors, even in patients with macroscopic PVTT [11, 12]. In order to avoid further tumor cell dissemination and reduce the stage of the tumor before surgery, we tested the efficacy of administering PIHP before surgery. Herein, we report a case of HCC involving PVTT for which a pathological complete response was achieved by the administration of PIHP with doxorubicin plus mitomycin C before surgery.

## Case presentation

**Fig. 1:**
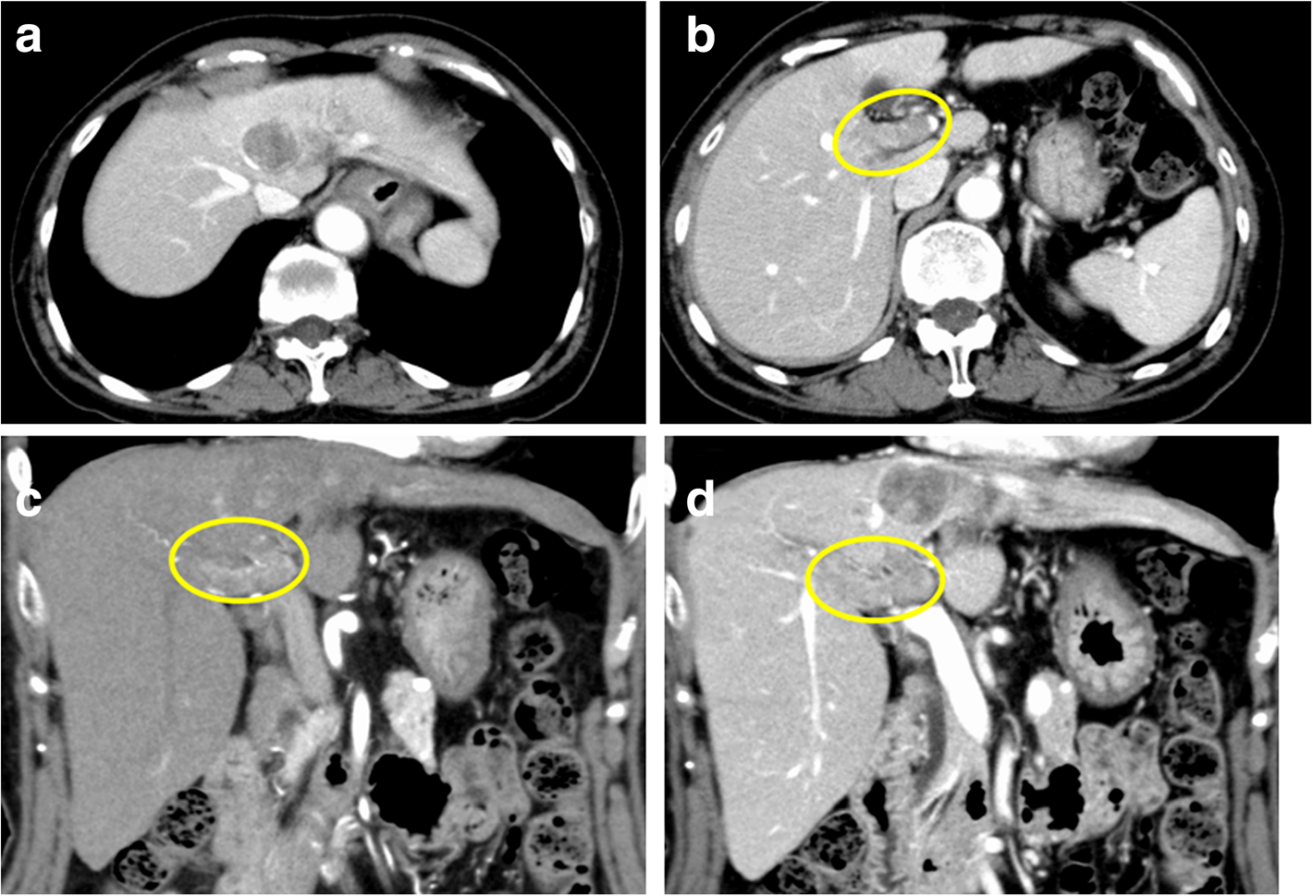
Contrast-enhanced CT before preoperative PIHP: CT showing a 28 × 25 mm HCC in segment 4 (**a**) and PVTT (*yellow circle*) in the left portal branch reaching the right portal (**b**). PVTT was enhanced in the arterial phase (**c**) and washed out in the equilibrium phase (**d**)

**Fig. 2:**
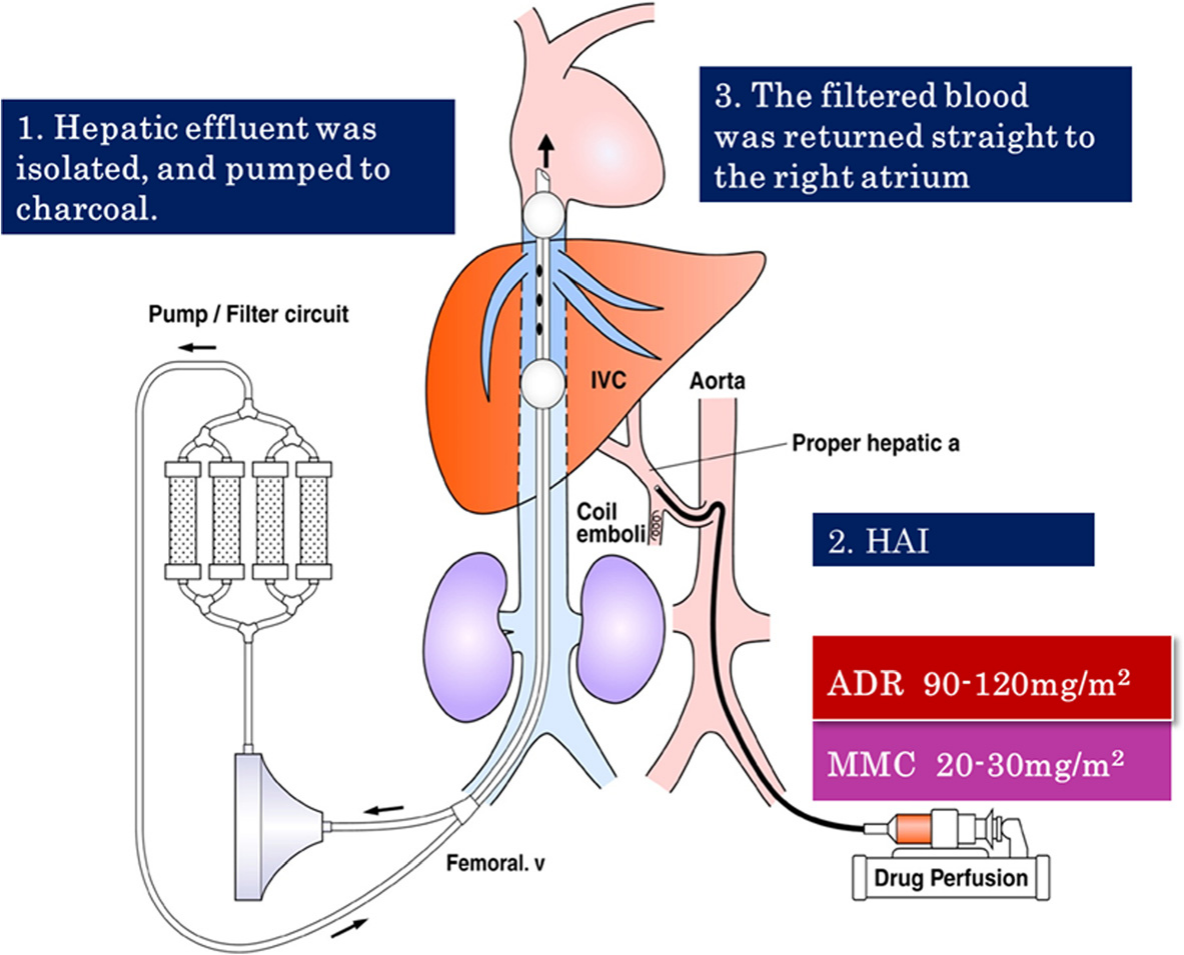
System of PIHP: The specially designed 4-lumen/2-balloon catheter was introduced into the retrohepatic inferior vena cava through the femoral vein. The hepatic effluent was isolated by balloon inflation and pumped to charcoal filters via the fenestrations of one major catheter lumen. The filtered blood was returned straight to the right atrium through the other major lumen with an opening at the distal tip of the catheter. The patient received a 30-min continuous HAI of 100 mg/m^2^ doxorubicin plus 30 mg/m^2^ mitomycin C. At the end of HAI, extracorporeal drug filtration was maintained at least for 10 min, and the 4-lumen/2-balloon catheter was removed

**Fig. 3:**
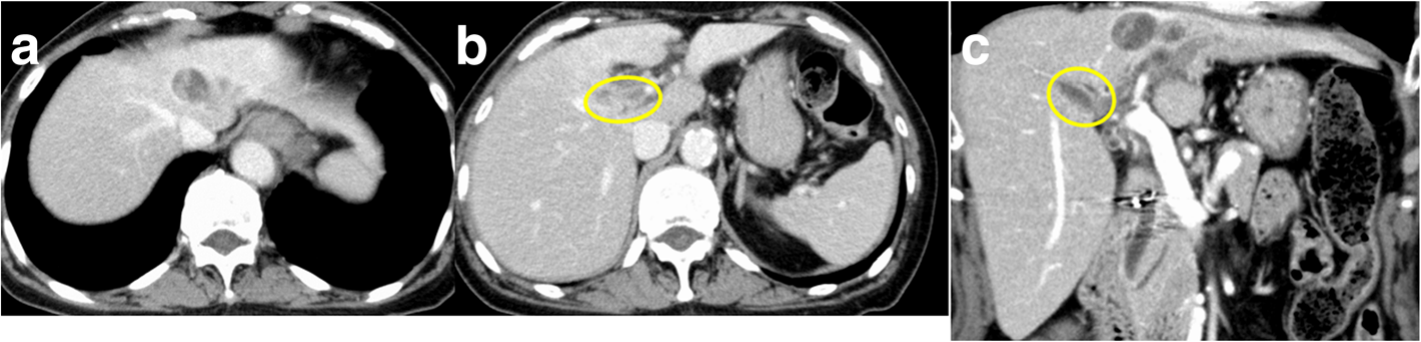
Contrast-enhanced CT after preoperative PIHP: CT showing the reduction of the main tumor volume (**a**) and no enhancement of the PVTT (*yellow circle*) after PIHP (**b**, **c**)

**Fig. 4:**
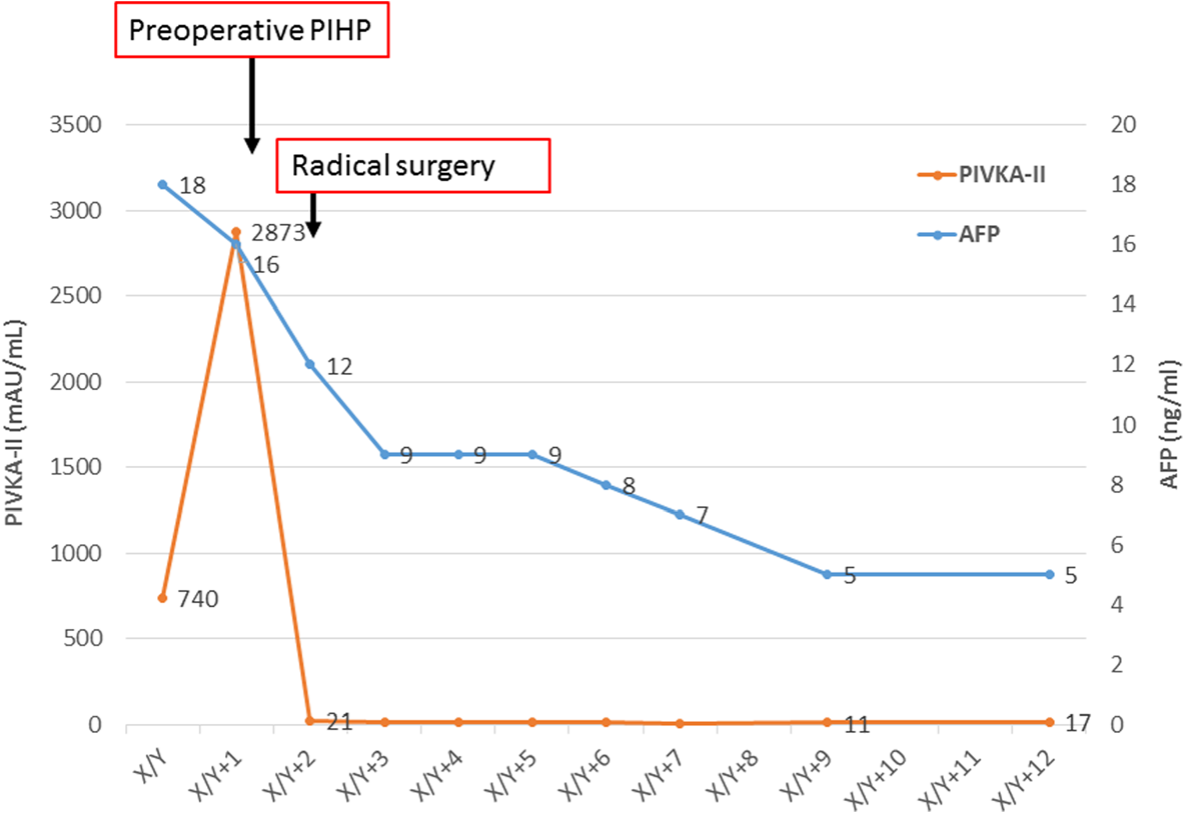
Perioperative clinical course: After preoperative PIHP, the serum level of AFP and PIVKA-II dramatically declined. She recovered uneventfully after the surgery and remained relapse-free 12 months after the surgery without any postoperative chemotherapy

A 77-year-old woman who was diagnosed with chronic hepatitis C at the age of 50 years was referred to a local hospital for follow-up. Abdominal ultrasonography showed a tumor in the left lobe of the liver. She was referred to our department for further examination and treatment. Laboratory data were as follows: platelet count 126 K/uL, total bilirubin 0.8 mg/dL, aspartate aminotransferase (AST) 176 U/L, alanine aminotransferase (ALT) 157 U/L, albumin 4.2 g/dL, prothrombin time 90.6 %, indocyanine green 15-min retention test (ICG R15) 19.7 %, α-fetoprotein (AFP) 16 ng/mL, protein induced by vitamin K absence or antagonist II (PIVKA-II) 2873 mAU/mL, hepatitis C virus (HCV) Ab positive, HCV RNA 6.54 logIU/mL. The liver damage was grade A and Child-Pugh classification was class A. Contrast-enhanced computed tomography (CT) showed a 28 × 25 mm tumor in segment 4, with a tumor thrombus in the left portal branch reaching the right portal branch, both of which were enhanced in the arterial phase and washed out in the equilibrium phase (Fig. 1). The patient was diagnosed with clinical stage T4 (Vp4) bN0M0, stage IVA HCC involving PVTT, according to the tumor-node-metastasis classification of the Liver Cancer Study Group in Japan (6th edition) [13], which indicated poor prognosis when treated with surgery only. After providing informed consent, the patient decided to receive PIHP before surgery. She received continuous hepatic arterial infusion (HAI) of 100 mg/m^2^ doxorubicin plus 30 mg/m^2^ mitomycin C for 30 min (Fig. 2). She recovered from a single dose of PIHP without severe toxicity, except a reduction in the grade 4 neutrophil counts (neutrophil count 200/μL) that was treated with recombinant G-CSF (CTCAE version 4.0) [14] on the 12th day after PIHP. The serum level of AST and ALT rose up to 516 U/L and 242 U/L on the 4th day after PIHP, respectively. CT showed reduction of tumor volume and no enhancement of the PVTT after PIHP (Fig. 3). The efficacy of chemotherapy was determined to be treatment efficacy (TE) 3 according to the criteria of the Liver Cancer Study Group in Japan (6th edition) [13] and based on the partial response achieved. The clinical stage after PIHP was determined to be T4N0M0, stage IVA. The serum levels of AFP and PIVKA-II dramatically declined to 16 ng/mL and 21 mAU/mL, respectively (Fig. 4). Subsequently, the neutrophil increased to 2400/μL, and the serum levels of AST/ALT improved to 36/61 U/L. The patient underwent extended left hepatic lobectomy and PVTT thrombectomy 6 weeks after PIHP. The operative findings revealed the extent of PVTT to be from the left and right portal branches to the portal trunk. Because the PVTT did not adhere to the portal vein wall, which would have led to a combined resection, she underwent thrombectomy with the back flow perfusion method and closure of the stump through a running suture of the portal vein with radical intent.

**Fig. 5:**
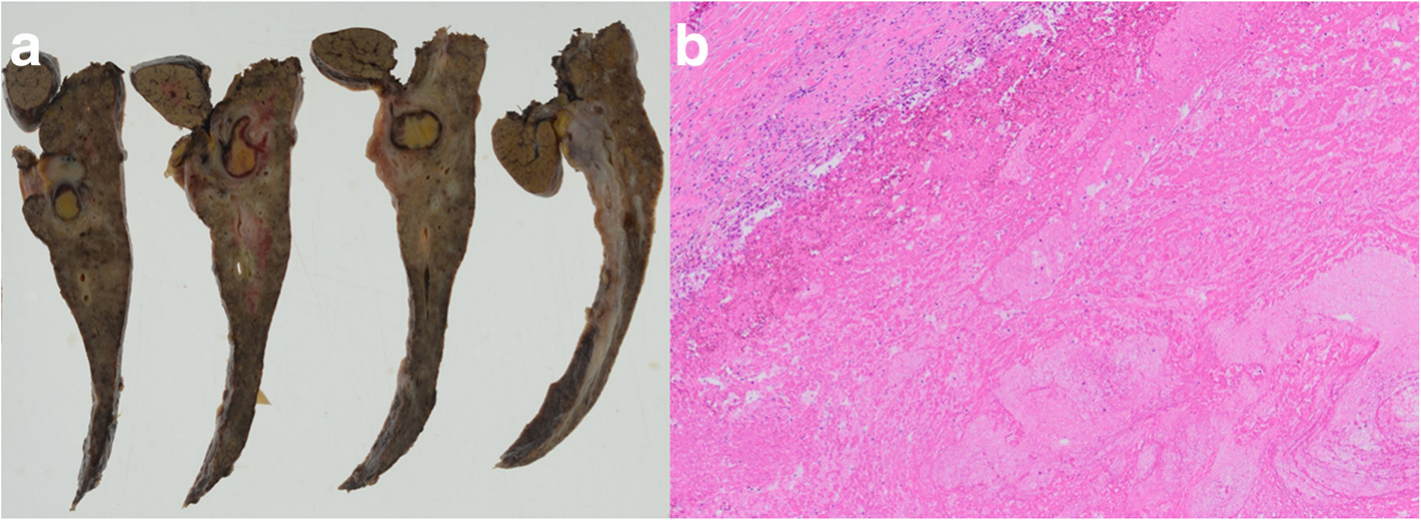
Macroscopic (**a**) and microscopic (**b**) findings on the resected specimen after PIHP: No residual tumor was found at the original site of the HCC, which extended into the portal branch in the left lobe of the liver. Instead, it was completely replaced by granulated and fibrous tissue. Microscopic findings in non-cancerous lesion showed grade 1 inflammation and stage 4/4 fibrosis with histopathologic features of chronic HCV. (hematoxylin and eosin stain, ×20)

Histological examination of the resected specimen revealed complete necrosis without any viable cancer cells in the primary tumor and PVTT (Fig. 5) and we further showed that treatment efficacy 4 and pathological complete response were achieved. The patient recovered uneventfully after surgery and remained relapse-free 12 months after the surgery, without any postoperative chemotherapy.

### Discussion

The optimal treatment method for patients with HCC involving PVTT in the portal bifurcation or the main trunk remains controversial [15]. PVTT represents one of the most important prognostic factors in patients with HCC. Most patients with HCC involving extensive PVTT have multiple intrahepatic metastases at the time of diagnosis and they are considered poor surgical candidates. If left untreated, patients with HCC involving PVTT have a median overall survival (OS) of 2–4 months [16, 17]. PVTT is also a causative factor for intrahepatic tumor dissemination, which primarily accounts for the poor prognosis in this group of patients [18, 19]. The most common staging system for HCC employed in American and European centers, the Barcelona Clinic Liver Cancer (BCLC) system, recommends against surgical resection in cases involving PVTT [20]. In a previous series of patients who underwent hepatectomy and thrombectomy for HCC involving PVTT in the main portal trunk, the 1- and 3-year OS rates were 25 and 4 %, respectively, and the disease-free survival rates were 3 % and 0 %, respectively [21]. However, surgical resection is considered the only curative option. To improve the efficacy of surgical resection for HCC involving PVTT, neoadjuvant/adjuvant therapies were evaluated in several studies [6, 22–24]. Because of the heterogeneous results, the effect of neoadjuvant therapy on the OS was inconclusive.

Since 1989, our center has been using PIHP for the treatment of advanced multiple HCC [6]. In our previous study, the data suggested a significant tumoricidal impact on less sensitive HCC and sufficient sorption with charcoal hemoperfusion of high-dose doxorubicin and MMC regimen [10]. In 2003, we reported the efficacy of reductive surgery plus PIHP for multiple HCC, including HCC with macroscopic PVTT [11]. The favorable survival profile suggested the efficacy of the second PIHP in local tumor control. Based on these previous reports, we extended the use of PIHP as preoperative therapy. In the present case, because the PVTT in the left portal branch reached the right portal branch and the high level of PIVKA-II indicated high recurrence possibility, surgery was planned after preoperative chemotherapy with PIHP. To the best of our knowledge, this is the first report of a case with pathological complete response after preoperative chemotherapy with PIHP in advanced HCC involving PVTT. Although there were several reports of spontaneous regression of PVTT [25, 26], in the present case, we judged PIHP was effective based on the pathohistological assessment.

Recently, the efficacy of preoperative HAI chemotherapy has been shown in patients with HCC and PVTT [27, 28]. Because PIHP has shown a high objective response rate (70.6 % complete and partial response) and safety, it might be a better alternative instead of preoperative therapy.

The clinical question of whether preoperative or postoperative PIHP is better should be addressed. Preoperative PIHP might not only reduce post-curative therapy recurrence but also downstage tumors from an unresectable to a resectable stage.

## Conclusions

In conclusion, further investigation into surgery following neoadjuvant chemotherapy as a possible therapeutic option for HCC involving PVTT is warranted. PIHP with doxorubicin plus mitomycin C is a feasible and promising preoperative chemotherapy for HCC involving PVTT.

## Consent

Written informed consent was obtained from the patient for publication of this case report and any accompanying images. A copy of the written consent is available for review by the Editor-in-Chief of this journal.

## Abbreviations

AFP, alpha-fetoprotein; ALT, alanine aminotransferase; AST, aspartate aminotransferase; BCLC, the Barcelona Clinic Liver Cancer; CT, computed tomography; HAI, hepatic arterial infusion; HCC, hepatocellular carcinoma; ICG R15, indocyanine green 15-min retention test; OS, overall survival; PIHP, percutaneous isolated hepatic perfusion; PIVKA-II, protein induced by vitamin K absence or antagonist II; PVTT, portal vein tumor thrombosis; TE, treatment efficacy
